# The Spatial Resolution of Epidemic Peaks

**DOI:** 10.1371/journal.pcbi.1003561

**Published:** 2014-04-10

**Authors:** Harriet L. Mills, Steven Riley

**Affiliations:** MRC Centre for Outbreak Analysis and Modelling, Department of Infectious Disease Epidemiology, Imperial College London, London, United Kingdom; University of Michigan and Howard Hughes Med. Inst., United States of America

## Abstract

The emergence of novel respiratory pathogens can challenge the capacity of key health care resources, such as intensive care units, that are constrained to serve only specific geographical populations. An ability to predict the magnitude and timing of peak incidence at the scale of a single large population would help to accurately assess the value of interventions designed to reduce that peak. However, current disease-dynamic theory does not provide a clear understanding of the relationship between: epidemic trajectories at the scale of interest (e.g. city); population mobility; and higher resolution spatial effects (e.g. transmission within small neighbourhoods). Here, we used a spatially-explicit stochastic meta-population model of arbitrary spatial resolution to determine the effect of resolution on model-derived epidemic trajectories. We simulated an influenza-like pathogen spreading across theoretical and actual population densities and varied our assumptions about mobility using Latin-Hypercube sampling. Even though, by design, cumulative attack rates were the same for all resolutions and mobilities, peak incidences were different. Clear thresholds existed for all tested populations, such that models with resolutions lower than the threshold substantially overestimated population-wide peak incidence. The effect of resolution was most important in populations which were of lower density and lower mobility. With the expectation of accurate spatial incidence datasets in the near future, our objective was to provide a framework for how to use these data correctly in a spatial meta-population model. Our results suggest that there is a fundamental spatial resolution for any pathogen-population pair. If underlying interactions between pathogens and spatially heterogeneous populations are represented at this resolution or higher, accurate predictions of peak incidence for city-scale epidemics are feasible.

## Author Summary

Fundamental spatial processes such as individuals' interactions and movement are not sufficiently well understood and yet they define the transmission of infectious diseases through populations. Spatial models of epidemics represent the region of interest (such as a city or country) as a collection of spatial units. To anticipate the magnitude and timing of peak incidence and to predict demand on health care resources in the region a clear understanding is needed of the relationship between the resolution of the representation (number and size of the pixels), the population interactions and the epidemic trajectories. We used a spatially explicit meta-population model of disease transmission to demonstrate that thresholds existed such that models with too low a resolution overestimated peak incidence, implying that ill-defined models may result in incorrect predictions. However, the results suggest that if population interactions are represented in sufficient detail, accurate estimates of peak demands on key health care resources are feasible.

## Introduction

Novel respiratory pathogens continue to pose substantial public health challenges, not least because of the risk that large epidemics may overwhelm key health care resources such as vaccination stockpiles and intensive care facilities. Recent epidemics of concern include: SARS [1], influenza [2]–[4], H7N9 [5], [6] and MERS [7], [8]. During an epidemic it is important to accurately predict the impact of the epidemic over different spatial scales, where scale refers to the size of the region being monitored; such as a hospital, city, country or globally. Intervention policies should be defined relative to this spatial scale, for example taking account of how long it will take to vaccinate a whole city or to distribute a treatment country-wide. Those making decisions about intervention strategies need a clear understanding of the underlying epidemic process, so as to anticipate the magnitude and timing of peak incidence at their scale of interest and to effectively control the epidemic.

Spatially explicit transmission models are used frequently to increase understanding of the spread of epidemics caused by pathogens which transmit between individuals close in space. For example: influenza [9]–[11], measles [12]–[14], and smallpox [15], [16] have all been represented by spatially explicit epidemic models. All of these examples can be thought of as metapopulation models in which the population of interest is represented as a collection of sub-populations located in space, for example households [17]–[19], airports (GLEaM [20]) or districts/states [21]. The advantages of these models are that they can capture complicated mobility and mixing patterns and heterogeneous population density, without the complexity of an individual-based model. Also, model output can be easily reported for specific populations, such as counties or cities.

It is known that heterogeneity both in population density and typical mixing behaviour heavily influence disease spread. Both of these are defined according to the resolution of the population representation, where resolution defines the number and size of the pixels making up the “image” of the population within the model. A pixel is the smallest single component of an image. A high resolution representation will divide the region into many small pixels; a lower resolution uses fewer, larger pixels (Fig. S1). The resolution chosen is usually decided by the data available: for example, population and travel data may be defined at the ward or county level only. The level of mixing between individuals in distinct pixels is defined by mobility models, these are often fitted to travel data from censuses. Also, sometimes, resolution is limited by computational capacity.

The concepts in our paper require precise definitions of the terms: scale, resolution and pixel. The literature using these three words is somewhat ambiguous with the terms resolution and scale sometimes used interchangeably. Therefore, for clarity, we have included explicit definitions at first use of the words (above) and in Table 1.

**Table 1:** Explicit definitions of the terminology used in this work.

Word	Definition
Scale	The relative size or extent of a region.
Resolution	The degree of detail visible in an image - in our work this defines the number and size of the pixels making up the representation of the region.
Pixel	The smallest single component of an image (or in our model, the smallest single component of the population representation).

## Results

**Figure 1:**
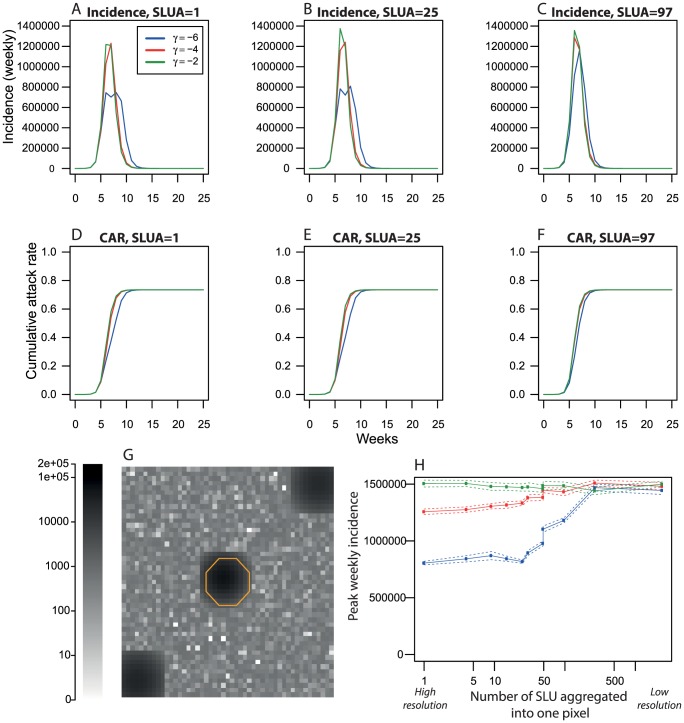
The effect of resolution and mobility in a theoretical population. A–C: Weekly incidence with time, for different spatial resolutions of the population. SLUA is the number of smallest LandScan units aggregated to make one pixel at that resolution. SLUA = 1 is the highest resolution possible in the data. D–E: Cumulative Attack Rate (CAR) for different resolutions of the population. Note that CAR is the same for all mobility levels and for all resolutions, but the epidemic trajectories differ. G: The population density of the theoretical population, the epidemic was seeded with 10 cases placed at random in the area indicated by the orange outline. H: Peak incidence for different levels of mobility (blue, very restrictive, green least restrictive). The number of smallest LandScan units aggregated to make one pixel increases from left to right along the x-axis (and resolution decreases). The spatial spread of the epidemic in these three scenarios is illustrated in a Movie S1.

We implemented a generic metapopulation model with arbitrary spatial resolution (see Methods) varying from approximately 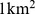 (30″ by 30″, the smallest unit representation) upwards (Fig. S1). We generated a theoretical population density in a region with total population just over 4 million and of size approximately 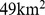 (49×49 pixels). The region had three ‘urban’ areas where population density was generated using a 2-dimensional bivariate Gaussian and a ‘rural’ area, generated from a uniform distribution, Fig. 1G. We used this formulation to simulate the spread of a pathogen representative of influenza, with an SIR-like natural history, assuming that the generation time was 2.6 days and the basic reproductive number 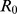 was 1.8. The epidemic was seeded with 10 individuals in a central region (Fig. 1G), simulations were repeated 25 times at each resolution.

The within-pixel contact rate was fixed for all pixels. Mobility between pixels was represented by a kernel with an offset power function. A kernel defines the relative probability of travelling between two pixels. The offset power function is an adaptation of the gravity model. The gravity model states that an individual's probability of mixing in a pixel different to their home pixel is inversely proportional to the distance apart of the pixels, to some power. The offset power function adds in an offset distance parameter, which means that pixels closer together than this distance mix fully. See Methods for a full definition of the kernel and the resulting mobility model.

Initially we considered three different kernels: we used an offset of 2 km and three different powers giving low, medium and high contact between pixels (the power, 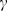, was −6, −4 and −2 respectively), Fig. S2. The highest mobility kernel is in line with kernels fitted to commuter data in the UK and US [9]. However, our review of data on travel patterns found that only 15% of an average individual's journeys are commuting, making up just 19% of the total distance an average individual travels each year [22] (Table S1). Commuting data also excludes key at-risk groups – the under 17 s and over 70 s – who have lower mobility travel patterns compared to the 18–69 population [22]. Therefore, we explored more restrictive kernels than those estimated using commuting data to reflect shorter distances travelled and lower frequency travel in the most at-risk populations and the regular non-commuting travel of the wider population.

We confirmed that the overall cumulative attack rate (CAR) for our model was independent of the mobility kernel and the model resolution (Fig. 1A–F). This was by design: the model was constructed such that with the assumption of mass action mobility (the rate of contact between two groups is proportional to the size of each of the groups) the epidemic was identical at every resolution. This means that the next generation matrix at any resolution and for any mobility has the same spectral radius: 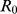 was the same at all resolutions and contact levels and the local and global 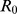
s were the same. A full proof that 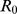 was constant with respect to resolution is given in the Text S1 in Supporting Information S1, and is similar to that in Ref [23]. Because 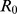 was constant, if the mobility was such that there was contact between every pair of pixels, the final epidemic size was the same across all resolutions and in every pixel. If mobility was restrictive enough that some pixels were never infected the final CAR reflected this restriction. The full proof that attack rates were constant with respect to resolution is in the Text S2 in Supporting Information S1, and is similar to those in Refs [24], [25].

For the theoretical population density, the existence of a fundamental spatial resolution was apparent: at resolutions lower than this threshold, system-wide peak incidence was substantially over-estimated, obtaining a high peak incidence and fast spread similar to that obtained in a fully mixed model (the lowest resolution). However, at the fundamental resolution and above, consistent estimates of the peak attack rate were obtained (Fig. 1H). This was increasingly evident as mobility became more and more restricted: for the most localised mobility assumptions (low power), peak incidence in the fully mixed case was nearly double that at the highest resolution. At high resolutions, multiple small pixels containing low numbers of individuals and with a high heterogeneity in population size slowed the epidemic spread; resulting in a long epidemic duration and a low peak incidence compared to low resolution model scenarios.

Increased mobility reduced the effect of resolution on the epidemic trajectory. At medium mobility, peak incidence increased with decreasing resolution but there was no distinct threshold. At the highest mobility, peak incidence was unaffected by resolution: the high level of contact between pixels facilitated the quick spread of the epidemic, indicated by a short epidemic duration and a high peak incidence at every resolution (Fig. 1A–C).

**Figure 2:**
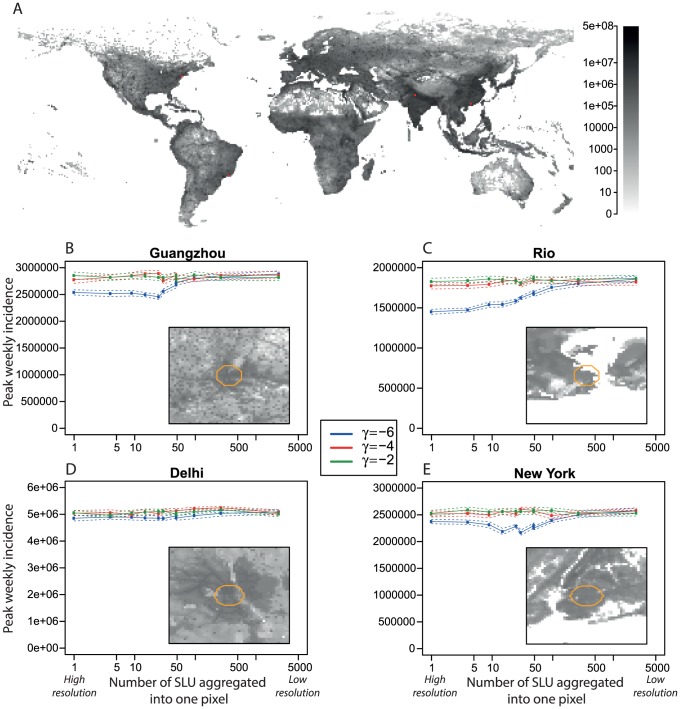
Results for actual populations from LandScan data[26]. SLU are the Smallest Landscan Units (30″ by 30″, or approximately 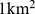). A: Population density of the whole world, the red spots indicate the locations of the 4 population densities we considered. The regions were chosen as a 49×49 unit square around their official lat-long centre. B–D: Main graphs indicate the peak incidence at each resolution, for Guangzhou (B), Rio (C), Delhi (D) and New York (E). The insets are the population densities of these regions, the colour scale is the same as panel A. The epidemics were seeded in a circle of radius 5 km around the official lat-long centre of the regions, the possible seeding area is marked by an orange outline on the insets.

Resolution and mobility remained important when the model was constructed with real population densities. We repeated the analysis (using the same three kernels) for four regions selected from LandScan data [26]: Guangzhou, Rio de Janeiro, Delhi and New York (Fig. 2A). The smallest LandScan unit is approximately 1 km^2^ (30″ by 30″) in size. The effect of resolution was most evident when mobility was more restricted, as with the theoretical population. In Guangzhou, Rio and New York, changing the spatial resolution had a significant effect on the peak incidence when mobility was at a low to medium level, though the effect was less clear in Delhi (Fig. 2B–E). The Delhi region had the largest total population size and the highest mean population density of all regions we considered (Table S2 and Fig. S3). Therefore, even at low mobility the numbers mixing will be relatively high, meaning that the disease spread will not be as restricted as it would be in a less densely populated region.

**Figure 3:**
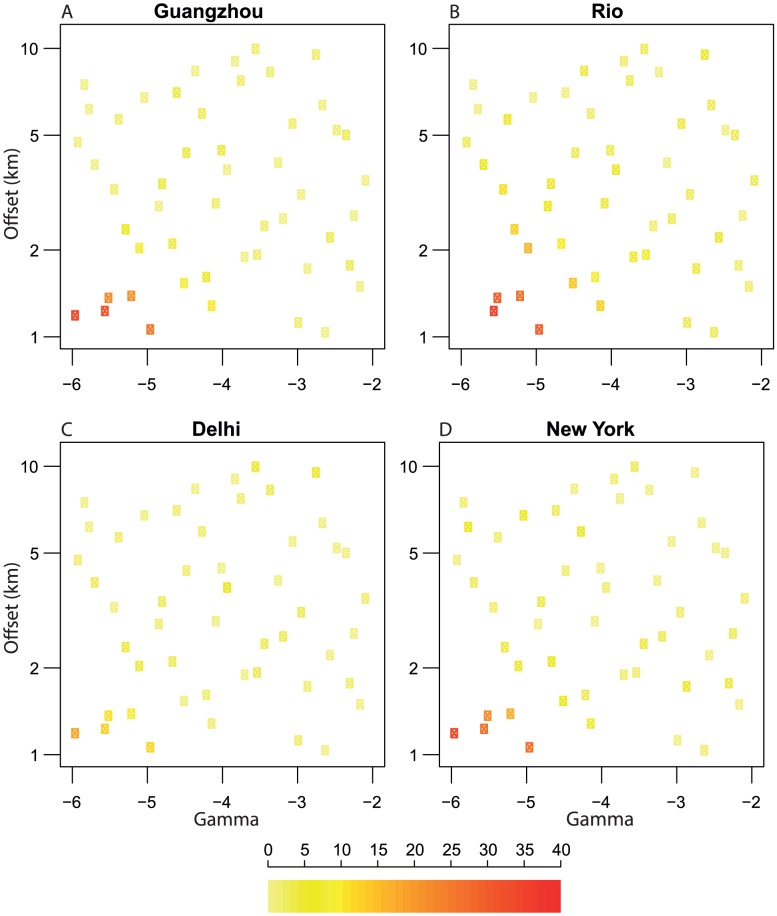
Latin Hypercube Sampling (LHS) results for four regions: Guangzhou, Rio, Delhi and New York. The LHS parameter sets varied 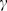 and the offset (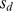) in the power law kernel, as described in the main text. We illustrate which parameter sets significantly affected the peak incidence at different resolutions by the colour of the point. The colour indicates the percentage change between the two points of lowest resolution and the two points of highest resolution, the colour-bar scale runs from 0% to 40%. Some parameter sets gave higher peak incidence at high resolution than low resolution (around −3% change), these were assumed to be caused by stochasticity as the number of runs was relatively small (10 repeats); the change for these was fixed at 0%. See Fig. S5 for plots of the trends in peak incidence for each parameter set.

We used Latin Hypercube Sampling (LHS) [27] to determine whether the patterns we saw with the illustrative mobility kernels could be generalised within a wider parameter space of mobility functions. We varied the kernel parameters: the power, 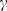, between −6 and −2 (as discussed earlier, this selection gave a wide range of mobility levels) and the saturation distance between 1 and 10 km, choosing from a log scale (so smaller distances are more likely). We tested 50 parameter sets chosen using the LHS technique [27], with 10 separate realisations of each set for each region (variation in results from the stochastic model was low - see confidence intervals for 25 repeats in Fig. 2 for example). Kernels for the 50 sets are plotted in Fig. S4. The LHS results confirmed that the effect of resolution is most important in populations which are less mobile, Fig. 3. As mobility decreased (a combination of the offset and the power in the kernel) the difference in peak incidence between the lowest and the highest resolution increased. This was particularly true in Guangzhou, Rio and New York, but in Delhi the effect was reduced (due to Delhi having a very large population in comparison to the other regions).

**Figure 4:**
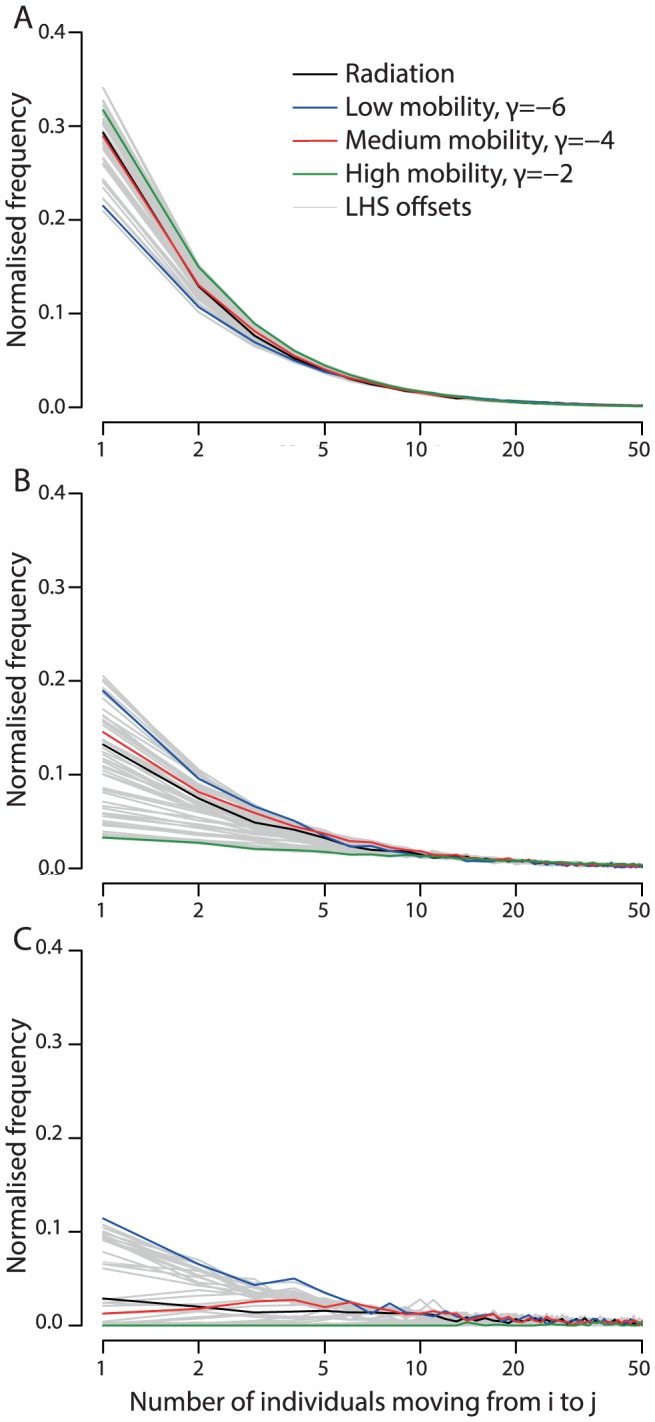
The number of individuals moving between populations (the flux) as defined by the offset gravity model (low, medium and high mobility and the LHS models) and for comparison the radiation model. We illustrate the mobility models at three resolutions: A: highest resolution, B: medium resolution and C: low resolution. The highest mobility model has higher average flux compared to the low and medium mobility models. At lower resolutions and high mobility large numbers of individuals move, but the distribution of the numbers moving is more uniform than at the highest resolution, causing the lines for the three mobility scenarios to swap order at different resolutions.

Recently it has been suggested that the movement of individuals depends not only on the source and destination cities, but also on the population density of the surrounding area [28]. This model is called the radiation model and has been proposed as a distinct alternative to the gravity model. However, we calculated the actual number of individuals moving between pixels (the flux) and found the radiation model flux to be very close to the offset gravity model of medium mobility, particularly at the highest resolution, Fig. 4. Indeed the radiation model is always bounded by the three gravity models we use and our LHS models explore a large space around these. More generally, gravity-like models have been implemented with a number of different normalisation assumptions, some of which produce population flux patterns very similar to the radiation model [15], [17].

## Discussion

When managing epidemics it is desirable to know the size and duration of the epidemic and the magnitude and timing of the peak incidence over the spatial scale of interest [4], [29], [30]. This scale of interest may be a city, a region or a whole country. Resources such as treatment, vaccinations and diagnostic tests will take time to be deployed over this scale and it can take time to develop and generate enough of these resources for the whole affected population [31], [32]. Accurate predictions about the magnitude and timing of peak incidence would greatly enhance the ability of public health officials to effectively limit the impact of epidemics.

We have shown how the representation of population interactions can impact model estimates of key epidemic outcomes. We examined the effect of the resolution of the population density on the model predictions of epidemic spread over the scale of interest. We refer to resolution as defining the number and size of the individual pixels dividing the region; higher resolution representations use a higher number of smaller pixels. Our results imply that for plausible population densities and mobility patterns, fundamental resolutions exist for specific pathogens such that the detail of the population and their interactions must be represented faithfully if accurate epidemic trajectories are to be estimated.

The impact of model resolution was clear in models of less mobile populations: our results indicate that at lower mobility, low resolution representations overestimated the peak incidence, obtaining a high peak incidence and fast spread similar to that obtained in a fully mixed model. However, sufficiently high resolution representations gave lower and later peak incidences because of the delaying effect of multiple small pixels. Indeed at low mobility, clear thresholds existed for the resolution of the theoretical population density, such that models with resolutions below the threshold over-estimated the system-wide peak incidence. Similar thresholds existed for real population densities: Guangzhou, Rio, New York and Delhi. Increased mobility reduced the effect of resolution on the epidemic.

The kernels which were most affected by resolution were those which gave a lower mobility than that identified by commuting data (Table S1). Generally children are considered to cause the majority of transmission of pathogens like flu and measles, because their level of age group assortative mixing is very high [33], [34]. Children also travel less far than working adults [22]. Together, these imply that a kernel for children is likely to be more restrictive than those defined by commuting data alone. Therefore, our results indicate that the correct specification of population interactions and sufficient spatial resolution is particularly relevant for epidemics such as measles and flu - those in which children play a large role.

Although we have considered age effects implicitly by including lower mobility levels than are reported for commuting data (Table S1), the explicit representation of age within a similar modelling framework may lead to additional insight. For example, transmission dynamics at different scales may be driven by different age groups: the behaviour of more mobile adults may be disproportionately important in the seeding of nearby pixels. However, the slower than expected within-country spatial spread during 2009 [35] suggests that for pandemic influenza, population sub-groups with reduced mobility likely do define the fundamental resolution.

We have chosen to represent the real biological process by a high resolution metapopulation model. Although we have not been able to push the model to resolutions higher than 1 km by 1 km, we suggest it is reasonable to assume, for the mobility kernels considered here, that the thresholds observed for peak incidence would not change substantially were we to approach the resolution of an individual-based model.

The model used here was intended specifically to test only the changing resolution of the disease transmission process. By design we did not want to assume that transmissibility was intrinsically higher or lower in different parts of the population. In future work, we hope to calibrate this model structure using actual disease incidence data and (after a minor modification to the definition of the force of infection) test for the possibility that population density affects transmissibility.

Although it is somewhat reassuring that estimates of peak incidence are biased upwards if resolution is too low, the epidemic duration is underestimated. In order to avoid the effects of incorrect model specification, where possible, spatial resolution should be treated in a similar manner to temporal resolution in fixed-time-step models: neither the doubling nor halving of spatial resolution should have a substantive effect on key model outputs.

## Methods

### Model description

We defined a spatially explicit meta-population model as follows (similar to Ref. [21]). A given region of known population density was represented as 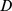 pixels, such that each pixel (index 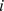) is the same spatial size but the number of individuals in the pixel (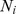) varied according to location. Mixing between and within each pixel was determined by a mobility model, represented by a matrix 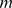 such that an entry 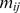 was equal to the probability that for an individual from pixel 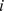, given that the individual made a contact, this contact was with an individual from pixel 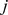 (mobility was defined using a kernel, discussed later).

The rate at which susceptible individuals in pixel 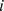 became infected depended on (1) their risk of infection from those in pixel 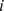, (2) the risk of infection from infected individuals in pixel 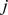 who travelled to 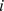, (3) the risk of infection that susceptible individuals from 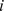 encountered when they travelled to 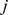. Therefore, the *force of infection* or the average rate that susceptible individuals in pixel 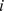 became infected per time-step was:
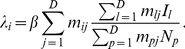
(1)where 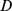 was the total number of pixels and for any pixel 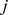, 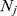 was the number of individuals, 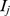 was the number of infected individuals and infectious contacts were made with other individuals present in the pixel with rate 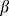. Note that 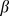 is the same across all pixels; in future it may be of interest to vary the transmissibility across pixels (so 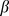 moves into the sum in Eqn. (1) as 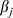).

The system of difference equations for a pixel 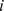 in the stochastic SIR model was (with the condition that all classes hold a whole number of individuals):
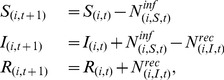
(2)where 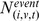 was the number of individuals in pixel 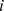, state 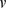 (S, I or R) that experienced the event – infection or recovery – in time-step 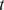. We ignored death in this model as we considered fairly short timescales and a non-fatal strain of influenza.

Each time-step 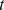, the number of individuals experiencing each event (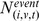) that occurred in pixel 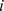 and state 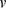, with a population 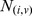 was determined in the following way:

1. For each pixel 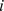, state 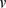, the probability that any event would happen to an individual in that pixel and state was:
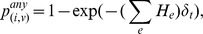
where the 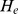 were the rates for the events that may occur in that compartment, (e.g. the rate of recovery, recall that these parameters were chosen to reflect the natural history of influenza).
2. For each pixel 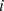, state 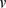, the total number of individuals who experienced an event 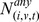 was chosen from a Binomial distribution,
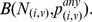

3. The numbers of individuals experiencing each event (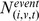) were drawn from a multinomial distribution with 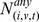 trials and the normalised selection probabilities 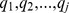 where
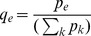


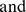


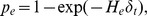
where 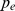 was the probability of each event occurring.

### Mobility model

We used a mobility model to determine the relative frequency of potentially infectious contact. This was represented as a matrix 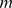 with entries 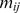, defined as the probability that for an individual from pixel 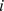, given that the individual made a contact, this contact was with an individual from pixel 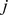, so:
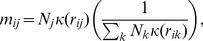
(3)where 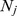 was the total population in pixel 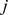, 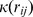 was the interaction kernel defining the effect of the distance between pixels 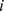 and 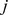 on the contact between them. The kernel defines the relative probability of travelling between two pixels and not the absolute flux, similar to [15], [17]. The factor 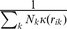 normalised 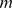 and ensured that the rows sum to 1. The matrix 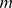 was used in the calculation of force of infection, Eqn. (1).

We used a variation of the offset power function for the kernel (similar to [9], [15]):
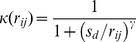
(4)where 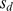 was the distance below which the kernel function saturated, we used 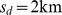. The power 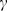 determined the mixing between pixels, this was varied to give a range of mobilities but was always less than 0.

### The next generation matrix and 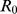


The next generation matrix, 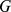, for the model with 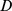 pixels and force of infection 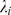 (Eqn. (1)), can be defined (similar to [21]):
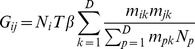
(5)where 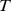 was the time spent infected (which depended on recovery rate 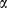 such that 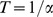, same for all pixels), 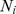 was the number of individuals in pixel 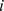, infectious contacts were made with other individuals present in the pixel with rate 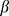 and 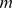 was the mobility matrix defined earlier. Then 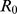 was equal to the spectral radius of this matrix 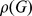 [36], [37]. For this model, 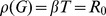, i.e. 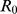 was independent of resolution and mobility; see Text S1 in Supporting Information S1 for full derivation.

## Supporting Information

Figure S1 **A simple example to illustrate how the resolution of the population is changed by aggregating multiple smaller units into larger units.** The raster package in R is used to manipulate the population data. A: The population at its finest spatial resolution, 36 squares total. B: Four (2×2) of the smallest units from the population in A are combined into one pixel. C: Nine (3×3) of the smallest units (from A) are combined to make one pixel. D: The whole region is considered as one pixel. Note that the populations of the aggregated units are summed to find the total number of individuals in the new pixels.
(EPS)

Figure S2
**The kernel function,** 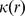 **against the distances,** 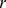 **, for the three different mobilities that we consider in the main results.** The kernel is an offset power law function, 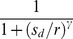, where 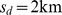 and 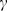 is −6, −4 and −2.(EPS)

Figure S3 **Details of the regions used in the main analysis.** Histograms of the population densities of the four regions: Guangzhou, Rio, Delhi and New York and unprojected spatial maps of the populations. The central point is marked by a red dot and the area where the epidemic was seeded is marked in orange.
(EPS)

Figure S4
**The kernels for the 50 parameter sets used in the Latin Hypercube Analysis of the model.** The kernel is an offset power law function defined in the main text. The parameter sets vary 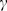 between −6 and −2 and 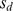 between 1 and 10 km (on a log scale).(EPS)

Figure S5
**The impact of resolution on peak incidence for a range of parameter sets chosen by Latin Hypercube Sampling.** The results are for four regions: Guangzhou, Rio, Delhi and New York. SLU are the Smallest Landscan Units (30″ by 30″ or approximately 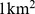). The LHS parameter sets varied 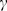 and the offset (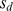) in the power law kernel, as described in the main text. The colour of the line indicates the percentage change between the two points of lowest resolution and the two points of highest resolution, the colour-bar scale runs from 0% to 40%. Some parameter sets gave higher peak incidence at high resolution than low resolution (around −3% change), these were assumed to be caused by stochasticity as the number of runs was relatively small and the change was fixed at 0%.(EPS)

Movie S1
**The spatial spread of the epidemic in a theoretical population for the three mobility scenarios considered in the main text.** From top to bottom is most restrictive (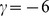) to least restrictive (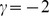). On the left: the spread of an epidemic seeded in the centre of the region, indicated by the prevalence. On the right: peak incidence in the region. These plots are for the highest resolution of the theoretical region described in Fig. 1 of the main text.(PDF)

Supporting Information S1 **The spatial resolution of epidemic peaks.** **Text S1:** Simplification of the next generation matrix. **Text S2:** Final epidemic size.
(PDF)

Table S1 **A review of current data and studies on mobility patterns in humans.** There is a lack of empirical data detailing why people travel, mode and distance travelled, divided by age and gender.
(PDF)

Table S2 **Details of the 4 regions used in the main paper.** All regions are 49×29 cells in size (2401 cells total), area varies according to latitude and longitude. Rio has a large number of zero regions because it is on the coast. Delhi has the highest population density but also the highest variance in population sizes. The map in Fig. 2 indicates the locations of these regions on the world map. Fig. S3 contains histograms of the population densities and spatial maps.
(PDF)
