# Supplementary material for: The Spatial Resolution of Epidemic Peaks
Source: PLoS Comput Biol. 2014 Apr 10;10(4):e1003561. doi: 10.1371/journal.pcbi.1003561 (PMC3983068; doi:10.1371/journal.pcbi.1003561)

Epidemic spreading across a theoretical population density

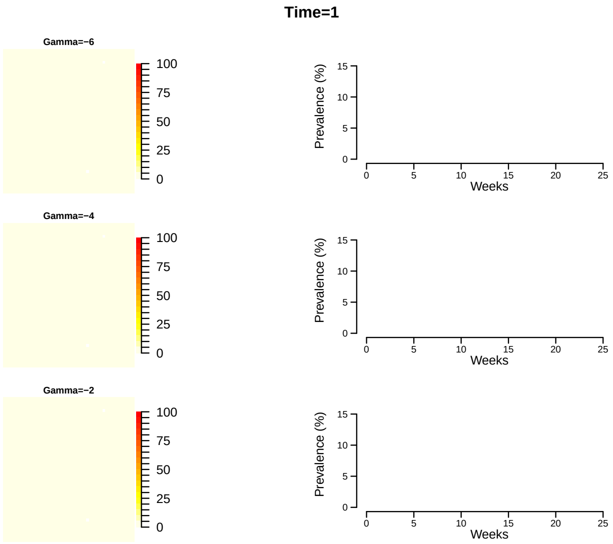

Supplement: Movie S1 — The spatial spread of the epidemic in a theoretical population for the three mobility scenarios considered in the main text. From top to bottom is most restrictive () to least restrictive (). On the left: the spread of an epidemic seeded in the centre of the region, indicated by the prevalence. On the right: peak incidence in the region. These plots are for the highest resolution of the theoretical region described in Fig. 1 of the main text. (PDF) [file pcbi.1003561.s006.pdf]
