# Supplementary material for: The Spatial Resolution of Epidemic Peaks
Source: PLoS Comput Biol. 2014 Apr 10;10(4):e1003561. doi: 10.1371/journal.pcbi.1003561 (PMC3983068; doi:10.1371/journal.pcbi.1003561)
Supplement: Supporting Information S1 — The spatial resolution of epidemic peaks. Text S1: Simplification of the next generation matrix. Text S2: Final epidemic size. (PDF) [file pcbi.1003561.s007.pdf]

Supplementary Text: The spatial resolution of epidemic peaks

Harriet L Mills [1] and Steven Riley[1]

[1]*MRC Centre for Outbreak Analysis and Modelling, Department of Infectious Disease Epidemiology, Imperial College London*

S1 Simplification of the next generation matrix

Under certain circumstances, we can find a simple expression for R_0 from the next generation matrix. The derivation is similar to the simplification of R_0 for the multi-city epidemic model described in Ref. [1]. For this we need the following theorems:

Theorem S1.1 *Let B be a n by n matrix with transpose $C = B^T$. Then B and C have the same eigenvalues. (Standard result).*

Theorem S1.2 *Let B be a n by n matrix with entries b_{ij} and spectral radius $\rho(B)$. Then:*

$$min(\sum_j b_{ij}) \leq \rho(B) \leq max(\sum_j b_{ij}).$$

i.e. $\rho(B)$ is bounded by the limits of the row sums of B, (standard result, see for example [2]).

Corollary S1.3 *Using Theorems 0.1 and 0.2, it is therefore true that for a matrix B with entries b_{ij} and spectral radius $\rho(B)$, $\rho(B)$ is bounded by the column sums of B:*

$$min(\sum_i b_{ij}) \leq \rho(B) \leq max(\sum_i b_{ij}).$$

Proof By Theorem 0.1 it is true that for the matrices C and B where $C = B^T$, the spectral radius of C and B is the same: $\rho(C) = \rho(B)$.
By Theorem 0.2 it is true that the spectral radius of matrix C is bounded by the limits of the row sums of matrix C:

$$\min(\sum_j c_{ij}) \leq \rho(C) \leq \max(\sum_j c_{ij}). \tag{S1}$$

As $C = B^T$ the row sums of matrix C are equivalent to the column sums of matrix B: $\min(\sum_j c_{ij}) = \min(\sum_i b_{ij})$.
Therefore it follows that Eqn. S1 may be equivalently written:

$$\min(\sum_i b_{ij}) \leq \rho(B) \leq \max(\sum_i b_{ij}).$$

∎

Therefore, we calculate the column sums for the Next Generation Matrix defined as:

$$G_{ij} = N_i T\beta \sum_{k=1}^{D} \frac{m_{ik} m_{jk}}{\sum_{p=1}^{D} m_{pk} N_p} \tag{S2}$$

where T was the time spent infected (which depended on recovery rate r such that $T = \frac{1}{r}$, same for all pixels), N_i was the number of individuals in pixel i, infectious contacts were made with other individuals present in the pixel with rate β and m was the mobility matrix defined earlier. We let $\sum_{k=1}^{D} m_{ki} N_k = N_i^N$, the total number of individuals in pixel i at any time (where m_{ki} is the probability that for an individual from pixel k, given that the individual made a contact, this contact was with an individual from pixel i). Recall that $\sum_{k=1}^{D} m_{jk} = 1$. Then summing the jth column of the next generation matrix gives:

$$\begin{aligned}
\sum_{i=1}^{D} G_{ij} =& G_{1j} + G_{2j} + ... + G_{Dj} \\
=& N_1 T\beta \frac{m_{11} m_{j1}}{N_1^N} + ... + N_1 T\beta \frac{m_{1D} m_{jD}}{N_D^N} \\
&+ N_2 T\beta \frac{m_{21} m_{j1}}{N_1^N} + ... + N_2 T\beta \frac{m_{2D} m_{jD}}{N_D^N} \\
&+ ... \\
&+ N_D T\beta \frac{m_{D1} m_{j1}}{N_1^N} + ... + N_D T\beta \frac{m_{DD} m_{jD}}{N_D^N} \\
=& \beta T m_{j1} \left(\frac{m_{11} N_1 + ... + m_{D1} N_D}{N_1^N} \right) \\
&+ ... \\
&+ \beta T m_{jD} \left(\frac{m_{1D} N_1 + ... + m_{DD} N_D}{N_D^N} \right) \\
=& \beta T (m_{j1} + ... + m_{jD}) \\
=& \beta T
\end{aligned} \tag{S3}$$

This holds for all columns j of G. Therefore using Corollary S1.3 it follows that $\rho(G) = \beta T$. This holds for all values of s_d and γ in the mobility matrix m and for any resolution (any number of pixels over the same region).

S2 Final epidemic size

We showed in the main text that the final epidemic size (the final cumulative attack rate) for a population was the same at all resolutions and all mobilities we considered (as long as for all pixels, i and j, $m_{ij} > 0$). Here we demonstrate how this is an outcome of the particular way our model was defined. The derivation is based on those in Refs. [3, 4].

Our SIR meta-population model can be defined as a system of differential equations as follows:

$$\begin{aligned}
\frac{dS_i}{dt} &= -\lambda_i S_i \\
\frac{dI_i}{dt} &= \lambda_i S_i - r I_i \\
\frac{dR_i}{dt} &= \gamma I_i,
\end{aligned} \tag{S4}$$

where r is the recovery rate and λ_i is the force of infection in pixel i:

$$\lambda_i = \beta \sum_{j=1}^{D} m_{ij} \frac{\sum_{l=1}^{D} m_{lj} I_l}{\sum_{p=1}^{D} m_{pj} N_p}. \tag{S5}$$

D is the total number of pixels, m is the mobility model and N_i is the total number of individuals in pixel i. Transmission occurs during a contact between an infected and a susceptible individual at a rate β.

Then it is straightforward to show that a final equilibrium exists, we represent this equilibrium as $S_i(\infty)$, $I_i(\infty)$ and $R_i(\infty)$ and we can show that $I_i(\infty) = 0$ and $S_i(\infty) + R_i(\infty) = N_i$.

We have shown earlier that R_0 for the system is constant and $R_0 = \beta T$, where $T = \frac{1}{r}$, the mean infectious period.

In each pixel i the fraction who did not get infected is $\sigma_i = \frac{S_i(\infty)}{S_i(0)}$, so the final epidemic size in each pixel is $x_i = N_i(1 - \sigma_i)$ (recall that N_i is the total population in pixel i). Then the total final epidemic size in the whole population is

$$Z = \frac{\sum_i^D x_i}{\sum_i^D N_i} = \frac{\sum_i^D N_i(1 - \sigma_i)}{\sum_i^D N_i}. \tag{S6}$$

We divide $\frac{dS_i}{dt}$ (Eqn. (S4)) by S_i and integrate between 0 and ∞, then we have:

$$\begin{aligned}
\int_0^\infty \frac{\frac{dS_i}{dt}}{S_i} dt &= \int_0^\infty -\lambda_i dt \\
&= -\int_0^\infty \beta \sum_{j=1}^{D} m_{ij} \frac{\sum_{l=1}^{D} m_{lj} I_l}{\sum_{p=1}^{D} m_{pj} N_p} \\
&= -\beta \sum_{j=1}^{D} m_{ij} \frac{\sum_{l=1}^{D} m_{lj} \int_0^\infty I_l dt}{\sum_{p=1}^{D} m_{pj} N_p}
\end{aligned} \tag{S7}$$

and we note that:

$$\begin{aligned}
\int_0^\infty \frac{dR_i}{dt} dt &= \int_0^\infty r I_i dt \\
R_i(\infty) - R_i(0) &= r \int_0^\infty I_i dt \\
&\text{therefore} \\
\int_0^\infty I_i dt &= \frac{R_i(\infty) - R_i(0)}{r} \\
&= \frac{N_i(1 - \sigma_i)}{r}
\end{aligned} \tag{S8}$$

as $R_i(\infty) - R_i(0) = N_i(1 - \sigma_i)$, the final epidemic size in pixel i. Therefore,

$$\int_0^\infty \frac{\frac{dS_i}{dt}}{S_i} dt = -\beta \sum_{j=1}^{D} m_{ij} \frac{\sum_{l=1}^{D} m_{lj} \frac{N_l(1-\sigma_l)}{r}}{\sum_{p=1}^{D} m_{pj} N_p} \tag{S9}$$

and as it is also true that:

$$\begin{aligned}
\int_0^\infty \frac{\frac{dS_i}{dt}}{S_i} dt &= log\left(\frac{S_i(\infty)}{log(S_i(0))}\right) \\
&= log(\sigma_i).
\end{aligned} \tag{S10}$$

we can write (recalling that $\frac{1}{r} = T$):

$$log(\sigma_i) = -\beta T \sum_{j=1}^{D} m_{ij} \frac{\sum_{l=1}^{D} m_{lj} N_l (1-\sigma_l)}{\sum_{p=1}^{D} m_{pj} N_p} \tag{S11}$$

equivalently, as $x_i = N_i(1-\sigma_i)$:

$$x_i = N_i \left[1 - \exp\left(-\beta T \sum_{j=1}^{D} m_{ij} \frac{\sum_{l=1}^{D} m_{lj} x_l}{\sum_{p=1}^{D} m_{pj} N_p} \right) \right]. \tag{S12}$$

Now, with the condition that $m_{ij} > 0$ for all i, j (i.e. there is always some contact between every pixel), then if the total final epidemic size is non-zero, then the final epidemic size in each pixel, x_i or $(1-\sigma_i)$, is also non-zero [3]. To see this, let an arbitrary pixel i have no infection, so $\sigma_i = 1$, then Eqn. (S11) implies that

$$\beta T \sum_{j=1}^{D} m_{ij} \frac{\sum_{l=1}^{D} m_{lj} N_l (1-\sigma_l)}{\sum_{p=1}^{D} m_{pj} N_p} = -log(\sigma_i) = log(1) = 0. \tag{S13}$$

But, as $m_{ij} > 0$, then for the left hand side to be zero it must be that $(1-\sigma_l) = 0$ for all l, so no pixel has infection. Therefore, if $Z > 0$ then $x_i > 0$ for all pixels i.

Now, we focus on the RHS of Eqn. (S11). Note that the matrix m (mobility) is a positive, right stochastic matrix, as its rows sum to 1. Therefore, $\rho(m) = 1$ (by definition of a stochastic matrix, and using Corollary S1.3).

We can simplify the RHS as follows (recall that $\sum_{p=1}^{D} m_{pj} N_p = N_j^N$:

$$\begin{aligned}
&-\beta T \sum_{j=1}^{D} m_{ij} \frac{\sum_{l=1}^{D} m_{lj} N_l (1-\sigma_l)}{\sum_{p=1}^{D} m_{pj} N_p} \\
&= -\beta T \sum_{l=1}^{D} \sum_{j=1}^{D} \frac{m_{ij} m_{lj} N_l (1-\sigma_l)}{N_j^N} \\
&= -\beta T \sum_{l=1}^{D} (1-\sigma_l) \left[\frac{m_{i1} m_{l1} N_l}{N_1^N} + ... + \frac{m_{iD} m_{lD} N_l}{N_D^N} \right] \\
&= -\beta T \Bigg[(1-\sigma_1) \left(\frac{m_{i1} m_{11} N_1}{N_1^N} + ... + \frac{m_{iD} m_{1D} N_1}{N_D^N} \right) + ... \\
&+ (1-\sigma_D) \left(\frac{m_{i1} m_{D1} N_D}{N_1^N} + ... + \frac{m_{iD} m_{DD} N_D}{N_D^N} \right) \Bigg]
\end{aligned} \tag{S14}$$

Therefore, let C be a D x D matrix, with entries $C_{ij} = -\beta T \sum_{l=1}^{D} \frac{m_{ij} m_{lj} N_l}{N_j^N}$, then the RHS of Eqn. (S11) is the ith row of matrix C multiplied by **(1-σ)**, which is a vector of length D with entries $(1-\sigma_j)$.

Note that the matrix C is mathematically similar to R, the next generation matrix (defined in Eqn. (S2)); this means that there exists a matrix Δ, such that $C = \Delta^{-1} R \Delta$. In this case, Δ is the D x D matrix with the number of individuals in each pixel, N_i, on its diagonal. As C and R are similar, they have the same spectral radius: $\rho(C) = \rho(R) = R_0 = \beta T$. We note that the row sums of C are all equal to βT, the proof is similar to that for the column sums of R in the next generation matrix simplification (i.e. note that $\sum_{j=1}^{D} \sum_{l=1}^{D} \frac{m_{ij} m_{lj} N_l}{N_j^N} = 1$).

Then, as all rows are equal, transmission in each patch is equal (to βT) and so, as in [3], the final fraction infected is given implicitly by $F = 1 - \exp(-R_0 F)$. With $R_0 = 1.8$ as in the main text, this can be solved numerically to give $F = 0.73243$, as we saw from the simulations.

References

[1] Arino J, Van den Driessche P (2003) A multi-city epidemic model. Mathematical Population Studies 10: 175–193.

[2] Cohen J (1979) Random evolutions and the spectral radius of a non-negative matrix. In: Mathematical Proceedings of the Cambridge Philosophical Society. Cambridge Univ Press, volume 86, pp. 345–350.

[3] Ma J, Earn D (2006) Generality of the final size formula for an epidemic of a newly invading infectious disease. Bulletin of Mathematical Biology 68: 679–702.

[4] Andreasen V (2011) The final size of an epidemic and its relation to the basic reproduction number. Bulletin of Mathematical Biology 73: 2305–2321.
