# Supplementary material for: The Spatial Resolution of Epidemic Peaks
Source: PLoS Comput Biol. 2014 Apr 10;10(4):e1003561. doi: 10.1371/journal.pcbi.1003561 (PMC3983068; doi:10.1371/journal.pcbi.1003561)
Supplement: Table S1 — A review of current data and studies on mobility patterns in humans. There is a lack of empirical data detailing why people travel, mode and distance travelled, divided by age and gender. (PDF) [file pcbi.1003561.s008.pdf]

Table S1: A review of current data and studies on mobility patterns in humans. There is a lack of empirical data detailing why people travel, mode and distance travelled, divided by age and gender.

Study or survey	Overview	Highlights
National Travel Survey (UK), 2011	UK based survey identifying personal travel patterns in Great Britain. Running since 1988. Approximately 20,000 individuals in 8,000 households take part each year. Collects data on how, why, when and where people travel. Data is split by age group and gender. 2011 data released in Dec. 2012; 2012 data will be available July 2013.	Results highlight the differences between the <17 and 70+ groups and the rest of the population - both in number of trips, purpose and distances. For example, 27% of trips that <17s make are for education - but these only make up 14% of total distance travelled. Over all age groups only 15% of the total average number of trips a year are commuting (19% of the total average distance). Even among the 18-69 yr group only 20% of journeys are commuting (23% of distance).
Truscott and Ferguson 2012 [1]	Informed by data from US and UK based census' on distances travelled for work. Uses piecewise kernels, including an offset power law, to represent the mobility. Runs an epidemic model to fit the kernels to a data driven network.	Considers three different resolutions, shows that higher resolution requires higher mobility to acquire the same epidemic. Model fitted from commuting data only. The kernels determined are very similar to the highest mobility kernel we use in the paper (but with different offsets and powers).
Brockmann et al 2006 [2]	Uses dollar bill tracking (the "Where is George?" campaign) to identify the distribution of distances travelled by humans.	The distribution decays as a power law (power=1.59). Other reviews have wondered if the observed distribution reflect all users of the note. Additionally, maybe the <17s and 70+ are unlikely to register the notes, biasing the data somewhat.
Gonzalez et al 2008 [3]	Uses mobile phone data from a random selection of 100,000 users to identify the distribution of distances travelled by humans. Location is recorded when a user sends or receives a text or call. Age distribution not specified.	Human movement according to this data shows high degrees of spatial and temporal regularity - people have consistent movement patterns. The distribution of displacements decays as a truncated power law, with a power of 1.75 (not far off that in the dollar bill study [2]).
Schneider et al 2013 [4]	Analysed mobile phone data over 154 days, in order to track individual's trajectories according to their call patterns.	Trip patterns created from mobile phone data were consistent with those created from travel surveys. Individuals had characteristic, reproducible trajectories (network motifs). Two western cities were compared and found very similar (Paris and Chicago). However, does mobile phone data capture all age group patterns? They could only use data from individuals whose mobile phone use was high enough to produce a nearly complete record of their locations.
Noulas et al 2012 [5]	Uses Foursquare data (a mobile app where users "check-in" to their location) to trace human displacement.	Using this dataset the distance covered by humans is determined by the number of places in between source and destination. The distribution of displacement decayed as a power law with exponent 1.50, similar to those previously identified. However, there is a possibility of strong demographic bias in the users of FourSquare.
Garske et al 2011 [6]	A large population survey of Shenzen city in Guangdong and Huangshan city in Anhui, China. Interested in variations in travel behaviour with age and gender. Commuting patterns (for work and education) were recorded in detail, but other travel was not so well documented.	They found that men travel further than women for work. Younger students travel less far to education (school) than older students. Commuting distances were very different in the two cities (e.g. Shenzen had a much higher drop off). Commuting was in general, much more local than in Western countries. Occasional travel was only recorded if the destination was outside the study area, so little was recorded.
Mossong et al 2008 [7]	Examined social contact patterns from 7,290 individuals of all age groups over one day. Particularly interested in type of contact and age group mixing.	Contact patterns were highly assortative with age, particularly among school children and young adults. Contacts at home, school or leisure were more often physical, compared to workplace contacts. The mixing pattern puts 5-19 yr olds at high risk of infection during an outbreak.

References

[1] Truscott J, Ferguson N (2012) Evaluating the adequacy of gravity models as a description of human mobility for epidemic modelling. PLoS Computational Biology 8: e1002699.

[2] Brockmann D, Hufnagel L, Geisel T (2006) The scaling laws of human travel. Nature 439: 462–465.

[3] Gonzalez MC, Hidalgo CA, Barabasi AL (2008) Understanding individual human mobility patterns. Nature 453: 779–782.

[4] Schneider CM, Belik V, Couronn T, Smoreda Z, Gonzlez MC (2013) Unravelling daily human mobility motifs. Journal of The Royal Society Interface 10.

[5] Noulas A, Scellato S, Lambiotte R, Pontil M, Mascolo C (2012) A tale of many cities: universal patterns in human urban mobility. PloS one 7: e37027.

[6] Garske T, Yu H, Peng Z, Ye M, Zhou H, et al. (2011) Travel patterns in china. PloS one 6: e16364.

[7] Mossong J, Hens N, Jit M, Beutels P, Auranen K, et al. (2008) Social contacts and mixing patterns relevant to the spread of infectious diseases. PLoS Med 5: e74.
