# Supplementary material for: The Spatial Resolution of Epidemic Peaks
Source: PLoS Comput Biol. 2014 Apr 10;10(4):e1003561. doi: 10.1371/journal.pcbi.1003561 (PMC3983068; doi:10.1371/journal.pcbi.1003561)
Supplement: Table S2 — Details of the 4 regions used in the main paper. All regions are 49×29 cells in size (2401 cells total), area varies according to latitude and longitude. Rio has a large number of zero regions because it is on the coast. Delhi has the highest population density but also the highest variance in population sizes. The map in Fig. 2 indicates the locations of these regions on the world map. Fig. S3 contains histograms of the population densities and spatial maps. (PDF) [file pcbi.1003561.s009.pdf]

Table S2: Details of the 4 regions used in the main paper. All regions are 49x29 cells in size (2401 cells total), area varies according to latitude and longitude. Rio has a large number of zero regions because it is on the coast. Delhi has the highest population density but also the highest variance in population sizes. The map in Fig. S2 indicates the locations of these regions on the world map. Fig. S3 contains histograms of the population densities and spatial maps.

Property	Guangzhou	Rio de Janeiro	Delhi	New York
Mean density	4,049	2,615	7,050	3,647
Max density	58,685	29,227	64,633	75,177
Variance	61,368,234	21,774,915	104,281,323	37,009,769
Number of zeros	5	1,258	2	824
Total population	9,722,479	6,280,196	16,926,573	8,755,909
Area (km^2)	1,900	1,903	1,814	1,567
Minimum Longitude	113.0583	-43.4	77.025	-74.14167
Maximum Longitude	113.4667	-42.99167	77.43333	-73.73333
Minimum Latitude	22.925	-23.11667	28.40833	40.46667
Maximum Latitude	23.33333	-22.70833	28.81667	40.875
